# Green and High Throughput Assay Using 96-Microwell Base to Determine Metformin Hydrochloride in the Tablet Dosage Form

**DOI:** 10.1155/2024/3374034

**Published:** 2024-08-23

**Authors:** Mohammed Alqarni, Atheer Alshehri, Bayan Almalki, Refah Althumali, Maram Alghamdi, Rawan Alqahtani, Safia G. Alotibi, Ali Alqarni, Adel H. Awad, Ibrahim A. Naguib

**Affiliations:** ^1^Department of Pharmaceutical Chemistry, College of Pharmacy, Taif University, P.O. Box 11099, Taif 21944, Saudi Arabia; ^2^College of Pharmacy, Taif University, P.O. Box 11099, Taif 21944, Saudi Arabia; ^3^Pharmaceutical Care Administration, Armed Forces Hospitals-Taif Region, P.O. Box 1347, Taif 21944, Saudi Arabia; ^4^Department of Oral & Maxillofacial Surgery and Diagnostic Sciences, Faculty of Dentistry, Taif University, P.O. Box 11099, Taif 21944, Saudi Arabia; ^5^Al Hada Armed Forces Hospital-Taif Region, P.O. Box 1347, Taif 21944, Saudi Arabia

## Abstract

Metformin (MET) is an oral antidiabetic drug widely used as the primary treatment for type 2 diabetes mellitus (T2DM). While various spectrophotometric assays exist for determining MET in pharmaceutical formulations, they often have limited throughput for quality control purposes. This study describes the validation of a 96-microwell plate spectrophotometer method using charge-transfer complexes (CTCs) with chloranilic acid (CLA) and 2,3-dichloro-5,6-dicyano-1,4-benzoquinone (DDQ) for the quality control and detected of MET. This reaction was carried out in 96-microwell plates, and the absorbance of the colored complexes of CLA and DDQ were measured at 530 nm and 460 nm, respectively, using an absorbance microplate reader. This study aims to identify and quantify the use of a 96-microwell plate spectrophotometer analytical technique for assessing complicated formulations. The method was successfully used for the quantification of MET in the tablet dosage form. The results showed good correlation coefficients (0.996 and 0.997) with CLA and DDQ, respectively. The present method showed high precision with RSD % not exceeding 2.17%. The accuracy of the method was obtained by recovery percentage, with percentage values less than ±5%. The Analytical Greenness Metric (AGREE) was used to evaluate greenness of the assays. The result show that the microwell assay method is greenness and suitable for handling large samples on a daily used with high throughput analysis. The use of the 96-microwell-plate method is superior to the existing method in terms of simplicity of the procedure, the low economic cost, and its consumption of low amounts of reagents and organic ethanol solvent, making it an environmentally friendly method. Therefore, these advantages make them suitable and rapid alternatives method to current methods for routine metformin analysis in quality control laboratories.

## 1. Introduction

Metformin, a synthetic biguanide, is an efficient and oral insulin-sensitizing antidiabetic drug that is used as the first-line antihyperglycemic for the treatment of type 2 diabetes mellitus (T2DM) in most patients. Typical diabetes mellitus complications include microvascular disorders such as diabetic kidney disease, retinopathy, peripheral and neuropathy, as well as macrovascular conditions including coronary heart disease, stroke, and peripheral arterial disease [1–5]. Since metformin is no longer under patent, it is also usually affordable and safe, with the most frequent adverse effects being gastrointestinal in nature and affecting 20–30% of patients [6]. Sometimes, unlabeled synthetic antidiabetic medications are illegally added to herbal remedies in the irresponsible pursuit of commercial goals. According to a laboratory investigation conducted by the US Food and Drug Administration, many herbal medications discovered in approved prescriptions that are used to treat diabetes contain active components that have not been disclosed. To track and assess these natural medications, it is crucial to create and validate suitable analytical techniques [7]. Quality control (QC) is an important process in the pharmaceutical sector and is necessary for drugs to be promoted as being clinically effective, safe to use, and therapeutically active. This ensures that consumers, physicians, and pharmacists have peace of mind that a given product will perform consistently and effectively for their intended purpose [8].

Numerous analytical techniques can be used to detect MET [9]. These techniques include UV-visible spectrophotometry [10–12], varied HPTLC techniques [13–15], HPLC [16, 17], LC-MS [18, 19], and spectrofluorimetric methods [20]. The high-performance liquid chromatography (HPLC) is a versatile tool for the quantification of pharmaceuticals while maintaining high levels of specificity and sensitivity [16, 17]. However, it has the problems of requiring higher level training, requiring a high-power source to operate, and it is expensive [21]. Liquid chromatography-mass spectrometry (LC-MS) is a combination of the ability to separate molecules through liquid chromatography and then identify the compound through mass spectrometry [18, 19].

To address the limitations of existing methods, alternative approaches must be explored and developed. This study aims to quantify the metformin in a complex formulation by using the 96-microwell plate spectrophotometer assay; charge transfer complexes were measured metformin with chloranilic acid (MET-CLA) and metformin with 2,3-dichloro-5,6-dicyano-1,4-benzoquinone (MET-DDQ). This method is characterized by simplicity, eliminating the need for trained chemists. It is also environmentally friendly as it reduces the volume of chemical substances required and has a high-throughput capacity, enabling the measurement of many samples with ease method. Moreover, it is cost-effective due to the decreased volumes used throughout the process.

## 2. Materials and Methods

### 2.1. Apparatus and Tools

All spectrophotometric analyses were performed using an absorbance microplate reader from Bio-Tek Instruments Inc., Winooski, United States. Gen5 software (Bio-Tek Instruments Inc. Winooski, USA) was used to control the reader. The UV-visible spectra were scanned using a double-beam ultraviolet-visible spectrophotometer (UV-1800, Shimadzu, Japan). We purchased clear 96-microwell plates from Corning/Costar Inc. in Cambridge, Massachusetts. The Sigma-Aldrich Chemicals Co. provided ethanol absolutes, and Dragon Lab provided adjustable pipettes.

### 2.2. Standards, Pharmaceutical Formulation, and Reagents

The MET hydrochloride raw material (Purity 99.6%) was a gift from Aljazerah Industry suppliers from the Auro Laboratories Company in India. There were two reagents used in this trial, namely, 2,3-dichloro-5,6-dicyano-1,4-benzoquinone (DDQ) from Tokyo Chemical Industry and chloranilic acid (CLA) and ethanol (analytical grade) both were purchased from Sigma-Aldrich.

### 2.3. Preparation of Standard and Sample Solution

Standard solutions: the 1 mg/mL stock solution from MET was prepared by dissolving with an accurately weighed amount of MET (10 mg) in 10 mL solvent (1 mL of water mixed with 9 mL of ethanol). This stock solution was stable for at least two weeks when kept in a refrigerator at 5°C.

Working solutions: the working solution was prepared from the standard solution by diluting it with ethanol to obtain different concentrations (from 50 *μ*g/mL to 250 *μ*g/mL) of metformin for analysis using the 96-microwell plate technique. Stock and working solutions were kept in a refrigerator at 5°C.

Pharmaceutical formulation sample solutions: ten tablets containing MET from different brands were accurately weighed and used as a fine powder. A quantity of the powder equivalent to 10 mg of MET was transferred into a measurement glass and then 1 mL of water and 9 mL of ethanol were added to the powder and mixed.

DDQ 0.5% was freshly prepared by weight (0.05 g from DDQ), and the mixture was diluted with 10 mL of ethanol and kept away from the light. The same technique was used for the CLA 0.5% reagent.

### 2.4. General Procedures

Each well of a 96-microwell plate was filled with an aliquot (100 *μ*L) of the standard or sample solution containing 10–100 *μ*L of MET, and 100 *μ*L of ethanol was added separately. Then, 100 *μ*L of DDQ or CLA solution (0.5% w/v) was added to each well, and the reaction was allowed to run for 5 minutes at room temperature (25 ± 2°C). An absorbance microplate reader was employed to assess the absorbencies of the resultant solutions at 460 and 530 nm, respectively.

### 2.5. Analytical Greenness Metric (AGREE)

The Analytical Greenness Metric (AGREE), a new system for evaluating greenness, is based on the twelve green analytical chemistry principles. The input criteria refer to the 12 SIGNIFICANCE principles [22], and the weights for each principle can be changed for greater flexibility. The sum of the ratings for each of the 12 input variables, which are translated into a score on a 0-1 scale, constitutes the final evaluation result [23]. The outcome is a clock-like circle with the total score and color image in the center [23, 24]. The segment's width reflects each concept's importance, and the red-yellow-green color scale denotes how effectively each principle is followed. A report and a graph are automatically generated after the assessment is finished using userfriendly, freely available software. By visiting the live link in the reference (http://www.mostwiedzy.pl/AGREE), AGREE software can be downloaded for free.

## 3. Results

### 3.1. Absorption Characteristics

**Figure 1:**
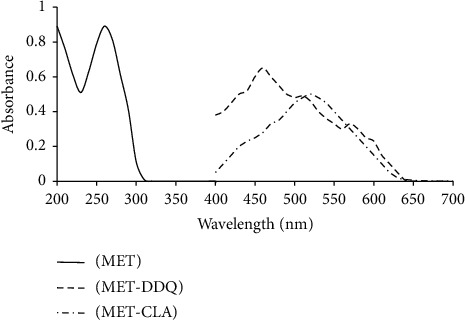
The absorption spectrum of metformin with and without each reagent in ethanol solutions on a single system without changing the measurement wavelength.

The UV-visible absorption spectra of the metformin solutions were obtained between 200 and 400 nm. The maximum absorption peaks were at 260 nm, and the minimum peaks were at 230 nm. Metformin solution was individually combined with each of the reagents CLA and DDQ, and the greatest peaks were 530 nm and 460 nm for the CLA and DDQ complexes, respectively. By adding the reagents CLA and DDQ, the UV-visible spectrum shifted in the range between 400 and 800 nm (Figure 1). Room temperature (25 ± 2°C) was maintained to continue the reactions.

### 3.2. Method Development

#### 3.2.1. Time Effect

**Figure 2:**
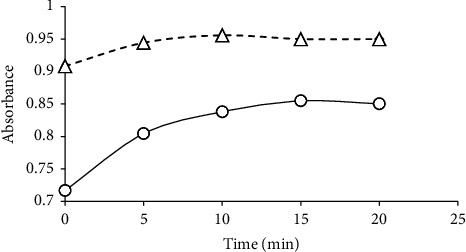
Effect of time on the respective C-T complexes' DDQ (—o—) and CLA (-- -Δ- - -) absorbance.

By observing the complex absorption at various time intervals after the addition of the CLA or DDQ solution to the MET solution, the times necessary to generate stable C-T complexes were measured. For CLA and DDQ, the color formation took 5 and 10 minutes to finish, respectively (Figure 2).

#### 3.2.2. Concentration Effect

**Figure 3:**
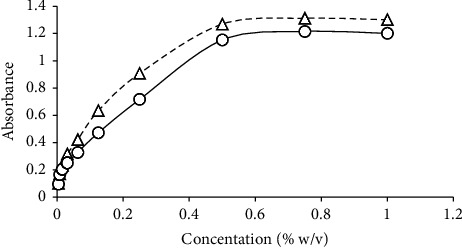
The effect of DDQ (—o—) and CLA (-- -Δ- - -) concentrations on each compound's MET complex.

The effects of the concentrations of CLA and DDQ on the corresponding complexes with MET are shown in (Figure 3). In this test, 10–100 *μ*L of 0.5% CLA or 10–100 *μ*L of 0.5% DDQ were each presented to MET for reaction. For the sample and the blank, the maximum and minimum values of absorbance were obtained.

#### 3.2.3. Job's Method

**Figure 4:**
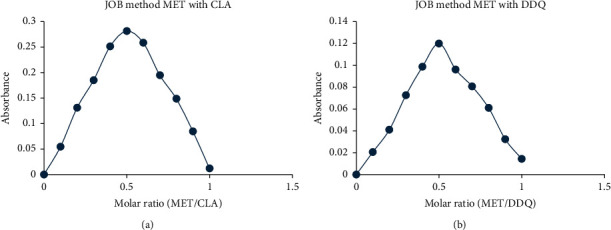
Job's plots in (a) CLA method and (b) DDQ method.

**Figure 5:**
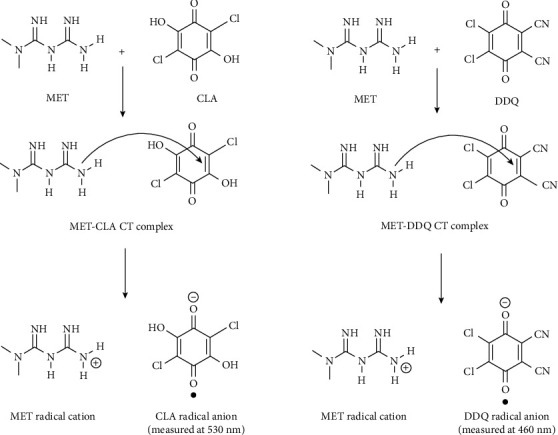
Mechanism of CT reaction of MET with CLA and DDQ.

To determine the stoichiometry of the reaction between metformin and either CLA or DDQ, the principle of Job's continuous variations approach was applied [25, 26]. In this experiment, 200 *μ*L of total volume was divided into equimolar solutions of medication and reagent. The maximum wavelength absorbance vs. the mole ratio of metformin was plotted for each technique. The MET-CLA and MET-DDQ C-T complexes' maximal absorbances and mole ratios (Figure 4) demonstrated that the MET medication and the reagent (CLA or DDQ) followed a 1 : 1 reaction stoichiometry [27]. Accordingly, the reaction was postulated to proceed as shown in Figure 5.

### 3.3. Validation of Proposed Assays

This method was validated according to ICH guideline [28].

#### 3.3.1. Linearity and Sensitivity

**Figure 6:**
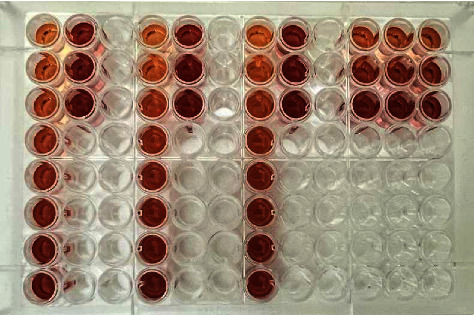
Image of the assay microplate of the proposed 96-microwell-based spectrophotometric assay of metformin.

**Figure 7:**
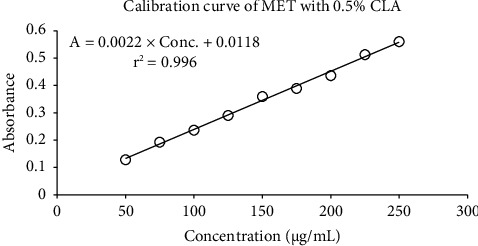
Linearity of MET with 0.5% CLA reagent.

**Figure 8:**
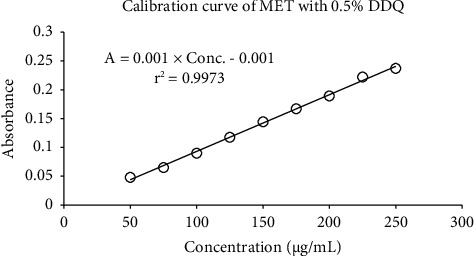
Linearity of MET with 0.5% DDQ reagent.

In 96-microwell plates, the optimal assay techniques were used to produce the color signals (Figure 6). The results of calibration graphs constructed for the determination of metformin via the reaction with CLA in 96-microwell plates are shown in Figure 7, followed by the reaction with DDQ in Figure 8. Each regression equation was calculated, and the outcomes are shown in Table 1. The obtained data demonstrate that the correlation coefficients (r2) for DDQ and CLA were 0.0996 for CLA and 0.997 for DDQ. The limits of quantification (LOQ) and limits of detection (LOD) were identified. In a 96-microwell plate, the LOD values for CLA and DDQ were found to be 18.12 *μ*g/mL and 15.17 *μ*g/mL, respectively. In a 96-microwell plate, the LOQ values for CLA and DDQ were 54.90 *μ*g/mL and 45.98 *μ*g/mL, respectively (Table 1).

The limit of detection (LOD) and limit of quantification (LOQ) were calculated from the standard deviation and its slope (SLOPE) as shown in the following equations:

(1) $$\begin{array}{c} \text{LOD}=\left(\frac{\text{SD}}{\text{SLOPE}}\right)*3.3,\\ \text{LOQ}=\left(\frac{\text{SD}}{\text{SLOPE}}\right)*10. \end{array}$$

#### 3.3.2. Precision and Accuracy

*(1) Precision*. Replicate analyses of the metformin solutions at three different concentration levels (50, 125, and 200 *μ*g/mL in each well) were assessed to evaluate the precision. Three repetitions of each sample from each test run were analyzed to determine the intra-assay precision, and three replicates from each assay run were analyzed to determine the interassay precision through different days (five days). The relative standard deviation (RSD) values were less than 2.17% for interday variations and 1.27% for intraday variations, which indicates great precision in terms of repeatability and only slight variance in the results (Table 2).

*(2) Accuracy*. Analytical recovery experiments were conducted to evaluate the assay's accuracy. The recovery percentage was measured using three replicates. The two assays' exceptional accuracy was demonstrated by their high recovery percentages (∼100%) with low SD values (Table 3).

(2) $$\begin{array}{c} \%\text{ Recovery}=\frac{MC}{TC}\times 100, \end{array}$$

where MC = measured concentration (calculated from calibration curve).

TC = theoretical concentration based on the weight

#### 3.3.3. Specificity

**Figure 9:**
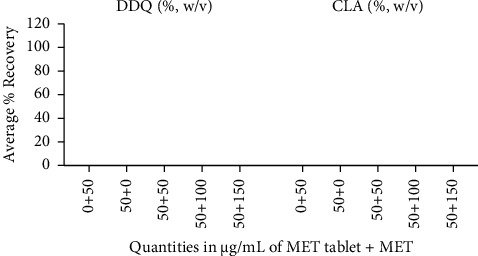
An illustration of the specificity of the proposed method for determining MET in the presence and absence of excipients using average percentage recovery. The quantities of MET were 0 + 50 (0 *μ*g/mL of MET tablet plus 50 *μ*g/mL of MET), 50 + 0 (50 *μ*g/mL of MET tablet plus 0 *μ*g/mL of MET), and 50 + 50 (50 *μ*g/mL of MET tablet plus 50 *μ*g/mL of MET, for a total MET concentration of 100 *μ*g/mL).

The specificity for the proposed method of MET determination in the presence and absence of excipients is presented in Table 4. 50 *μ*g/mL of MET tablet contain excipient was mixed with different concentrations of pure MET and analyzed for % recovery of MET. The average percentage recoveries ranged from 99.13% to 101.12% for MET-CLA and from 98.98% to 102.15% for MET-DDQ, which are within the accepted limit (∼100%) (Figure 9). These results demonstrate that the proposed method accurately quantifies MET in the presence and absence of excipients, confirming the specificity of the proposed method.

#### 3.3.4. Robustness

The robustness of the proposed method for MET determination was assessed by evaluating the average recovery percentages under various experimental conditions, including variations in reaction times and reagent concentrations. The results indicated that the average recovery percentages remained within the acceptable limit of approximately 100% (Table 5) and were unaffected by the changes in conditions. These findings demonstrate that the proposed method shows high precision and accuracy through different experimental conditions, confirming its robustness for MET determination.

#### 3.3.5. Determination of Metformin in Pharmaceutical Formulations

The previously validated methodologies were applied to analyze the commercially available MET pharmaceutical formulations, and a statistical comparison of the results was carried out between the calculated and theoretical values. Using three different concentrations of MET hydrochloride from a local pharmacy, i.e., Omformin® 500 mg, Formit® 750 mg, and Metformin hexal® 1000 mg, high recovery percentages (∼100%) were obtained which indicated their effectiveness (Table 6).

### 3.4. Greenness Assessment

**Figure 10:**
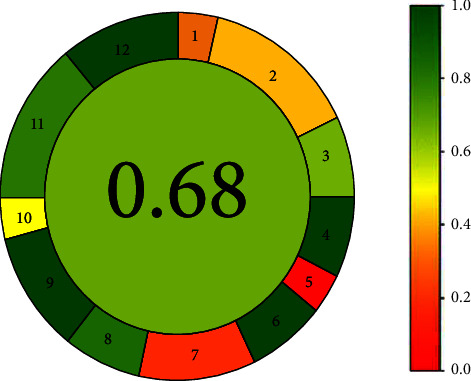
An illustration of AGREE analysis, with the circle denoting the evaluation's outcome on the left and the reference color scale on the right.

Greenness assessment results using AGREE tool resulted in a score value of 0.68, as shown in Figure 10 [23, 24]. The results reflect acceptable greenness of the applied microwell assay method, which favors the use of the proposed check method for large samples and daily use.

## 4. Discussion

Researchers have reported using several techniques to determine the quantity of MET [9–12, 15–17, 19, 27]. Analysis techniques such as UV-visible spectrophotometry, chromatography, and spectrofluorimetric methods have been referred to as selective MET methods. However, they have disadvantages because assay methods require expensive equipment and a highly skilled operator. Meanwhile, the reported ultraviolet spectrophotometric methods are similarly restricted by factors such as a shorter analytical wavelength where a photometric error may occur, lower sensitivity, limited linear range, or additional extraction processes using organic solvents [27]. In this method, with complete validation to assess and quantify metformin in pharmaceuticals using a 96-microwell plate spectrophotometer, C-T complexes were measured MET-CLA and MET-DDQ, and the color formation took 5 and 10 minutes. The reaction stoichiometry ratio of the MET medication and the reagent (CLA or DDQ) was measured at 1 : 1. The reaction is indicated by the following equation:

(3) $$\begin{array}{c} D^{\ldots }+A⟶\left[D^{\ldots }-A\right]\overset{\text{Polar solvent }}{⟶}D^{+}+A^{-}DA\text{ complex Radical ion}. \end{array}$$

The calibration curves over the concentration were linear with high correlation coefficients. The proposed method demonstrated high precision and accuracy with low RSD and high recovery, respectively. Compared with a recent study conducted in 2020 into the application of two charge-transfer complex reactions for selective determination of metformin hydrochloride in pharmaceuticals and urine, there are some similarities in the current results; for instance, the measurable wavelengths obtained were 530 nm and 460 nm at maxima [27]. Furthermore, that study applied spiked human urine analysis, which is useful for the physiotherapeutic administration of the drug. The benefit of the current study reduces the amount of organic solvent required with low costs, thus protecting the environment. Consequently, the proposed analytical method demonstrates suitability for routine quantitative analysis of metformin in both bulk substances and tablet dosage forms.

### 4.1. Assessment of High Throughputs of the Assays

According to the previous research [29–34], it was found that these microwell assays are way better than the traditional assays. This method stands out due to its simplicity, which eliminates the requirement for chemists with advanced training. It boasts environmental benefits by minimizing the volumes of chemical substances needed for samples, reagents, and waste management. Consequently, it also has a high-throughput capacity, allowing for the facile analysis of a considerable number of samples (around 600 samples/hour). In addition, it is economically viable due to the diminishment of chemical substance volumes utilized in the process. These assays have a high throughput feature, and if they were fully automated, the productivity would be even greater.

## 5. Conclusions

The conclusions of this study highlight the critical importance of recognizing and addressing the QA of drugs in the pharmaceutical industry, such as metformin, the necessity of stringent quality control procedures and regulatory oversight to guarantee the safety and efficacy of these medications. The reliable and precise analytical method presented in this study offers a useful tool for locating and selecting the active pharmaceutical ingredients in complicated metformin formulations, which can help researchers to assess the manufacture and distribution of metformin in tablet form. To address effectively this important public health concern, consumers, healthcare providers, and regulatory organizations must remain attentive. The 96-microwell-plate spectrophotometer method was based on the color production of C-T complexes with DDQ and CLA reagents. The use of the 96-microwell-plate method is superior to the existing method in terms of the simplicity of the procedure, the low economic cost of analytical reagents, and its consumption of low amounts of reagents and organic solvent (making it an environmentally friendly and green method). Thus, it can be applied to many samples in a short period. Its further advantages include the capacity of application in a high-throughput system and its ability to detect metformin using a single system without altering the detection wavelength. These benefits promote the use of this method for metformin analysis in quality control laboratories as an alternative to the current approach.

## Figures and Tables

**Table 1:** Quantitative parameter of linearity.

Parameter	96-microwell plate spectroscopy	
	DDQ method	CLA method
Reagent (%, w/v)	0.5	0.5
Linear range (*μ*g/mL)	50–250	50–250
Intercept	0.001	0.0118
Slope	0.001	0.0022
Correlation coefficient (*r*^2^)	0.997	0.996
LOD (*μ*g/mL)	15.17	18.12
LOQ (*μ*g/mL)	45.98	54.90

**Table 2:** Metformin HCL intra- and interday assay precision data (*n* = 3).

Method	Theoretical concentration (*μ*g/mL)	RSD %	
		Intraday	Interday
DDQ reagent	50	1.19	1.19
	125	1.27	1.68
	200	0.52	2.17

CLA reagent	50	0.93	1.42
	125	0.21	0.57
	200	0.35	0.46

**Table 3:** Metformin HCL % recovery studies and SD (*n* = 3).

Method	Concentration (*μ*g/mL)	% Recovery (average)	SD
DDQ reagent	50	100.59	1.20
	125	99.06	1.66
	200	99.72	2.17

CLA reagent	50	97.71	1.38
	125	97.78	0.55
	200	98.10	0.45

**Table 4:** Specificity of the proposed method of MET in present and absent excipients (*n* = 3).

MET tablet (with excipients) (*μ*g/mL)	Add MET (*μ*g/mL)	Total MET tablet + MET (*μ*g/mL)	Average recovery % (±SD)
*DDQ (%, w/v)*			
0	50	50	100.59 (1.20)
50	0	50	99.20 (1.20)
50	50	100	101.03 (1.65)
50	100	150	98.98 (1.71)
50	150	200	102.15 (0.99)

*CLA (%, w/v)*			
0	50	50	100.7 (0.90)
50	0	50	100.13 (1.38)
50	50	100	99.13 (2.21)
50	100	150	100.81 (0.97)
50	150	200	101.12 (1.31)

**Table 5:** The robustness of the proposed method for the determination of MET (*n* = 3).

Parameter		Average recovery (% ± SD)
DDQ (%, w/v)		
No variation		100.45 (1.27)
Reagent conc. (%, w/v)	0.6	99.06 (1.27)
	0.7	98.07 (0.95)
Reaction time (min)	8	100.29 (1.23)
	10	101.08 (1.84)

*CLA (%, w/v)*		
No variation		99.11 (0.21)
Reagent conc. (%, w/v)	0.6	101.88 (1.08)
	0.7	98.75 (1.37)
Reaction time (min)	8	99.11 (0.75)
	10	102.25 (1.66)

**Table 6:** Metformin HCL in commercially available pharmaceutical formulations ® data (*n* = 3).

Method	Commercially available pharmaceutical formulations ® (mg)	Average theoretical Concentration of MET (*μ*g/mL)	Average measured conc. of MET (*μ*g/mL), (SD)	Average recovery (%)
DDQ reagent	500	50	49 (2.08)	99.89
		100	99 (1.58)	99.20
		200	200.01 (0.79)	100.24
	750	50	49 (2.04)	99.20
		100	99 (0.6)	99.55
		200	202 (1.08)	101.11
	1000	50	49 (1.2)	99.20
		100	99 (1.04)	99.89
		200	202 (0.52)	100.93

CLA reagent	500	50	49 (1.38)	98.32
		100	102 (0.94)	101.69
		200	196 (0.35)	97.80
	750	50	50.5 (0.91)	100.73
		100	100.02 (0.94)	100.49
		200	196 (0.57)	98.18
	1000	50	50.5 (2.28)	101.03
		100	99 (1.19)	99.73
		200	198 (0.92)	98.86

## Data Availability

The data used to support the findings of this study are included within the article.
