# Corrigendum to “Green and High Throughput Assay Using 96-Microwell Base to Determine Metformin Hydrochloride in the Tablet Dosage Form”

**DOI:** 10.1155/ianc/9782127

**Published:** 2025-09-17

M. Alqarni, A. Alshehri, B. Almalki, et al., “Green and High Throughput Assay Using 96-Microwell Base to Determine Metformin Hydrochloride in the Tablet Dosage Form,” *International Journal of Analytical Chemistry* 2024 (2024): 3374034, https://doi.org/10.1155/2024/3374034.

In the article, there is an error in Figure 1 where the graph is blank, introduced during the production process. The correct Figure 1 is shown below:

We apologize for this error.

## Figures and Tables

**Figure 1:**
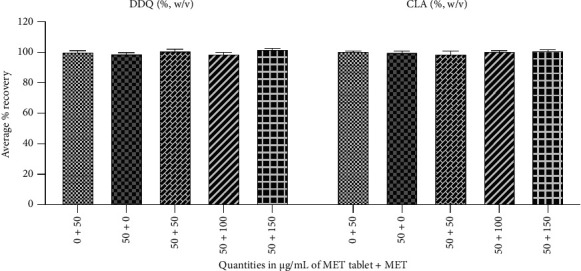
An illustration of the specificity of the proposed method for determining MET in the presence and absence of excipients, using average percentage recovery. The quantities of MET were 0 + 50 (0 μg/mL of MET tablet plus 50 μg/mL of MET), 50 + 0 (50 μg/mL of MET tablet plus 0 μg/mL of MET), and 50 + 50 (50 μg/mL of MET tablet plus 50 μg/mL of MET, for a total MET concentration of 100 μg/mL).

